# Lagos Bat Virus, an Under-Reported Rabies-Related Lyssavirus

**DOI:** 10.3390/v13040576

**Published:** 2021-03-29

**Authors:** Jessica Coertse, Marike Geldenhuys, Kevin le Roux, Wanda Markotter

**Affiliations:** 1 Centre for Emerging Zoonotic and Parasitic Diseases, National Institute for Communicable Diseases of the National Health Laboratory Services, Sandringham 2192, South Africa; jessicac@nicd.ac.za; 2 Centre for Viral Zoonoses, Department of Medical Virology, Faculty of Health Sciences, University of Pretoria, Pretoria 0001, South Africa; marike.geldenhuys@up.ac.za; 3 Epidemiology Unit, Allerton Veterinary Laboratory, Pietermaritzburg, KwaZulu-Natal 3200, South Africa; Kevin.LeRoux@kzndard.gov.za

**Keywords:** Lagos bat virus, lyssavirus, bats, rabies, Africa, South Africa

## Abstract

Lagos bat virus (LBV), one of the 17 accepted viral species of the *Lyssavirus* genus, was the first rabies-related virus described in 1956. This virus is endemic to the African continent and is rarely encountered. There are currently four lineages, although the observed genetic diversity exceeds existing lyssavirus species demarcation criteria. Several exposures to rabid bats infected with LBV have been reported; however, no known human cases have been reported to date. This review provides the history of LBV and summarizes previous knowledge as well as new detections. Genetic diversity, pathogenesis and prevention are re-evaluated and discussed.

## 1. Introduction

Until the 1950s, it was thought that rabies virus (RABV) was the only causative agent of the disease known as rabies, but this changed when the first rabies-related virus, Lagos bat virus (LBV), was detected in 1956 in Nigeria [1]. With improvements in diagnostic techniques and molecular characterization technologies, the known lyssavirus species have significantly expanded [2], and 17 species are now recognized within the *Lyssavirus* genus (Table 1) [3]. Of these, at least seven have been reported from Africa [4]. These species are grouped into two phylogroups and several ungrouped viruses based on antigenic and phylogenetic properties, with LBV belonging to phylogroup II. The diversity described from Africa has led to the hypothesis that lyssaviruses may have originated from the African continent and subsequently co-evolved with their primary hosts, i.e., bats, for thousands of years [5,6]. We discuss the history of LBV detections, serological surveillance and new detections (*n* = 8) from passive surveillance in South Africa. The genetic diversity is re-evaluated, and the pathogenicity and implications for prevention and control are discussed.

## 2. Lagos Bat Virus Detections

**Figure 1:**
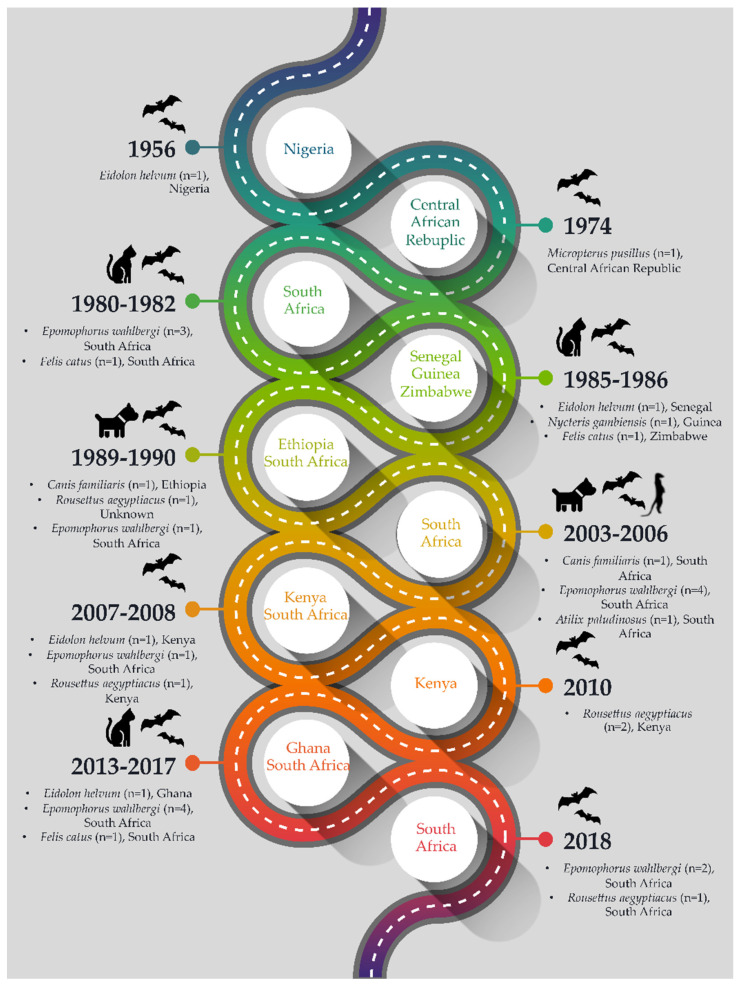
Timeline of laboratory confirmed Lagos bat virus detections.

Surveillance efforts focused on encephalitic and tropical fevers isolated a virus from male *Eidolon helvum* bats in 1956 on Lagos Island in Nigeria via the mouse inoculation test. This virus, LBV, was not neutralized by any rabies immune sera, indicating that it was not a strain of RABV [1] and was initially classified as a potential arbovirus. In 1969, members of the Yale Arbovirus Research Unit, in collaboration with the Centers for Disease Control and Prevention, USA, were able to classify LBV as a rhabdovirus using electron microscopy and showed that LBV was serologically related to another lyssavirus, i.e., Mokola virus (MOKV) only identified in the 1970s [7,8]. During the period 1956–2020, only 32 LBV cases were laboratory confirmed using monoclonal antibody typing or sequencing (Figure 1).

### 2.1. Detections in Eidolon helvum

A total of four LBV detections have been reported. After the first detection in 1956, LBV was only detected in *E. helvum* again 29 years later in Senegal [9]. A pilot study was conducted in Kenya during the period 2006–2007 in the Global Disease Detection Program framework to detect emerging infectious agents in bats. During this period, 1221 bats representing at least 30 bat species were tested, and one LBV isolate was obtained from a dead *E. helvum* bat. Several organs were also positive for viral RNA, including the brain, salivary glands, tongue, bladder, lung, stomach, adrenal glands, liver, heart, ovaries and kidneys [10]. In 2013, a broad spectrum non-targeted virus isolation study was undertaken in Kumasi, Ghana, and LBV was isolated from an apparent healthy *E. helvum* bat [11].

### 2.2. Detections in Epomophorus wahlbergi

All LBV detections in *Epomophorus wahlbergi* have been restricted to the KwaZulu-Natal (KZN) province in South Africa and were first detected in the early 1980s [12]. The province experienced an epidemic of canine rabies during this period, and heightened public awareness led to the submission of 282 bats noted for abnormal behaviour (fluttering on the ground) for rabies diagnosis. Ten bats submitted in 1980 and three in 1981 were positive for lyssavirus antigens with the fluorescent antibody test (FAT). Of these positive bats, only one was positively identified as *E. wahlbergi*, although it is believed that all individuals were of the same species. Of the 13 positive bats, only three were subsequently isolated and confirmed to be LBV with monoclonal antibody tests [9,12,13]. The next LBV case was reported in an *E. wahlbergi* bat found dead in 1990 [9]. LBV was only detected in *E. wahlbergi* again over a decade later in 2003 after implementing a passive surveillance study [14] during which four cases were identified [14,15]. In 2003, a dead *E. wahlbergi* was submitted for rabies testing after being caught by a domestic cat [14]. In 2004, a resident found a dead *E. wahlbergi* bat on her lawn one morning. She reported hearing squeaking noises around her house the previous evening. The FAT was positive for both bats with subsequent successful virus isolation, monoclonal antibody typing, RT-PCR and sequencing [14]. The following year (2005), the caretaker at a communal outdoor sports complex found an adult *E. wahlbergi* bat with her pup still attached on the lawn. The caretaker placed the bats in a nearby tree. However, they were later found on the ground again, where a cat was toying with them. The bats were then submitted to a local bat rehabilitator, though the adult bat had died. The FAT performed for the adult bat was negative; however, RT-PCR detected LBV RNA in the brain [14]. The pup had one apparent bite wound, presumably from the cat, but appeared to be healthy at submission to the bat rehabilitator and was feeding well, though it died four days later. Diagnostic tests (FAT and RT-PCR) on the pup’s brain material were negative [14]. An *E. wahlbergi* bat was submitted for rabies diagnosis from a bat rehabilitator in 2006. The bat was initially very calm but appeared to be dehydrated and had trouble breathing. The bat was given water and liquidized fruit; although the bat drank the liquids, the bat rehabilitator noted that the throat was inflamed with severe swelling. After consumption of the liquids, the bat started producing excessive amounts of saliva and shaking its head. The eyes of the bat appeared to be opaque, and its breathing deteriorated. The bat eventually started choking and died shortly after. This bat’s brain material was positive with FAT and identified as LBV with RT-PCR and sequencing [15,16]. Shortly after that, in 2008, LBV was detected in a euthanized *E. wahlbergi* bat submitted for rabies diagnosis by a local veterinarian. The veterinarian initially treated the bat for hyperthermia; however, the veterinarian decided to euthanize the bat after it started to display neurological signs, including difficulty swallowing [17].

### 2.3. Detections in Rousettus sp.

In 1999, a bat imported from Africa (possibly Togo or Egypt) died in the department du Gard, France, and was shown to be infected with LBV. The bat species involved was initially reported as *Pteropus* sp. but was later corrected to more likely be *Rousettus* sp. [18,19]. During the period 2008–2011, LBV was detected three times in *Rousettus aegyptiacus* in Kenya [20,21]. All three bats were found dead at different locations in Kenya, with nearly all tissues tested containing viral RNA. In addition, the virus could be isolated from a single faecal swab, and a significantly high viral titre of 10^7.5^ MICLD_50_ (i.e., the median lethal dose for mice inoculated by the intracerebral route) was detected in a salivary gland [20,22].

### 2.4. Detection in Micropterus pusillus

The first and only detection of LBV in *Micropterus pusillus* (current taxonomic status: *Epomophorus pusillus* [23]) occurred in 1974 from Bozo, Central African Republic. The virus was detected in the brain, heart and spleen [24]. It was indicated that, serologically, this isolate could be distinguished from the original LBV isolate from Nigeria, and that cross-reactions with MOKV antibodies occurred [25].

### 2.5. Detection in Nycteris gambianus

Lagos bat virus was detected for the first and only time in an insectivorous bat (*Nycteris gambianus*) during a survey in 1985 in Guinea [9].

### 2.6. Detections in Spillover Hosts

The first report of LBV in a spillover host (an individual that comes into contact with the reservoir and is infected [26]) was in 1982 from a domestic cat in South Africa (KZN province). The cat was behaving abnormally and was previously vaccinated [9,27]. Another vaccinated domestic cat tested positive for LBV in Zimbabwe in 1986 [28]. The cat from the mining village of Dorowa was submitted for routine rabies diagnosis in Harare. The female cat was euthanized after a three-day illness characterized by incontinence, hypersensitivity, posterior paresis, convulsions, excessive salivation and aggression when handled. The cat had been vaccinated against rabies with Rabisin^®^ three times, at three years, fifteen months and three months, prior to its LBV infection [28]. During routine rabies diagnosis performed at the National Research Institute of Health in Addis Ababa, Ethiopia, during the period 1989–1990, LBV was isolated from a domestic dog displaying rabies symptoms [29]. In 2003, LBV was detected in an Australian cattle dog that attacked people on a beach (Richards Bay, KZN). The dog was vaccinated against rabies; however, the owner was unsure of the vaccination date [15]. The first identification of LBV from a terrestrial wildlife species occurred in 2004 from a water mongoose (*Atilax palidinosus*) [30]. The animal was captured by the Society for the Prevention of Cruelty to Animals from a marshy valley in a residential area near Durban (KZN province, RSA). The animal appeared disorientated, attacked inanimate objects and alternated between friendly and aggressive behaviour.

## 3. New Lagos Bat Virus Cases Identified in South Africa, 2013–2018

Through passive surveillance networks, which include national diagnostic laboratories, bat rehabilitation centres, and bat interest groups, eight new cases of LBV have been identified in South Africa since 2013. Except for one case in *R. aegyptiacus*, all cases occurred in the KZN province of South Africa.

A large male *E. wahlbergi* bat was found grounded at a private residence in Scottburgh by children in 2013. They first thought that the bat was dead, but they provided the bat with water after discovering that it was alive. It was noted that the bat behaved abnormally. A local bat rehabilitator collected the bat, and it was observed that the bat was listless and had lost the use of its back legs. The bat’s condition deteriorated (clinical signs included fever, anorexia, opaque eyes and paralysis), but it continued to drink water. The bat died shortly after that. The brain material tested positive with FAT and quantitative real-time reverse-transcriptase polymerase chain reaction (qRT-PCR) [31] (laboratory identification number: UP2250).

During a small retrospective study performed to molecularly characterize FAT-positive samples from cats in KZN [32], LBV was detected (coordinates: −30.218130, 30.790770). In October 2013, the cat’s owner received bites from the animal when it was approached and subsequently took the cat to a local veterinarian. The veterinarian placed the animal in quarantine and observed clinical signs consistent with rabies, including aggression, anorexia and ataxia. The cat eventually lost all motor control and died approximately two weeks later. Brain material was submitted for rabies diagnosis and tested positive with FAT and qRT-PCR [31] (Allerton Provincial Veterinary Laboratory identification number: 13/599). Due to the bite sustained by the owner, post-exposure prophylaxis was initiated [33].

In January 2014, a juvenile *E. wahlbergi* was submitted to a veterinarian by a resident (coordinates: −29.812870, 30.862760). The veterinarian and a veterinary nurse at the clinic cared for the bat for approximately two weeks before it suddenly died. During this time, the bat appeared healthy, with no clinical signs of illness. The veterinary nurse was also bitten on the finger by the bat, and as such, post-exposure prophylaxis was initiated. The bat carcass was submitted for rabies diagnosis, and brain material tested positive with FAT and qRT-PCR [31] (Allerton Provincial Veterinary Laboratory identification number: 14/070). Several other organs were also positive for viral RNA with qRT-PCR, including the heart (1.76 × 10^4^ RNA copies), intestine (1.62 × 10^4^ RNA copies) and the salivary glands (5.62 × 10^3^ RNA copies) [33].

In April 2016, an adult male *E. wahlbergi* bat was found on a path (under palm trees, next to an electric fence) by children in Umhlanga. The bat was submitted to a local veterinarian and appeared to be healthy. However, due to the human exposures involved, post-exposure prophylaxis was initiated, and the bat was euthanized and submitted for rabies diagnosis. The brain material tested positive with FAT and qRT-PCR [31] (laboratory identification number: UP6414).

During the same time, another male *E. wahlbergi* bat was found in the Botanic Gardens (coordinates: −29.607437, 30.341093). The bat was submitted to a local bat rehabilitator that noted a pneumonia-like disease. The bat died approximately one month later and tested positive with FAT and qRT-PCR [31] (laboratory reference number: UP7398).

In March 2018, an adult female *E. wahlbergi* bat was submitted by a local veterinarian for rabies testing (coordinates: −30.084770, 30.865550). The reported clinical signs included profuse salivation, panting, general weakness, teeth grinding and the inability to open its eyes. The brain material tested positive for viral RNA with qRT-PCR [34] (laboratory reference number: UP8873).

A month later, a homeowner reported two fruit bats falling out of a tree on his property (coordinates: −30.08430, 30.86557). His two dogs attempted to retrieve the bats, during which one of the dogs received a bite from one of the bats. Both bats were submitted for rabies testing. One of these bats tested positive for viral RNA with qRT-PCR [34] (laboratory reference number: UP9878; Allerton Provincial Laboratory reference number: 18/495).

During the same time, an adult female *R. aegyptiacus* bat was found dead at Matlapitsi cave, GaMafefe, Limpopo province (coordinates: −24.11487, 30.12151), when sampled as part of a broader biosurveillance program on viruses. The brain material tested positive for viral RNA with qRT-PCR [34] (laboratory reference number: UP8931).

## 4. Surveillance for Lagos Bat Virus

**Figure 2:**
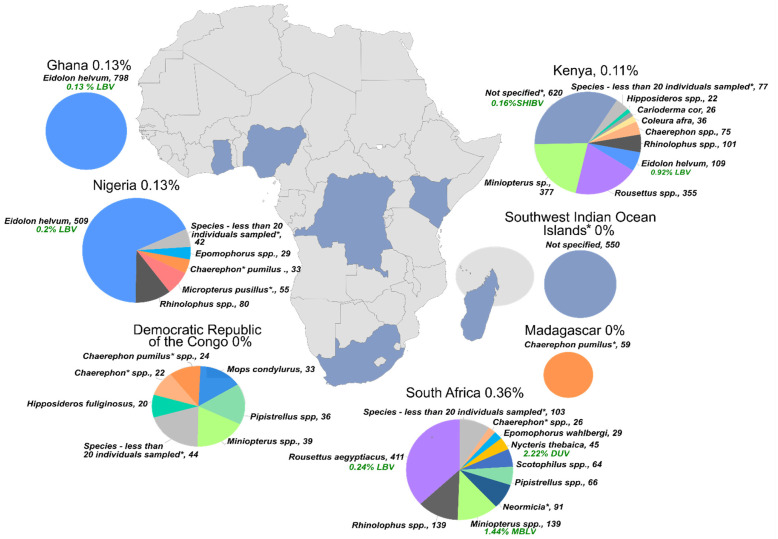
The number of bats surveyed for lyssavirus RNA during active nucleic acid surveillance activities depicted as pie charts. The countries in Africa where the surveillance was conducted are indicated in blue on the map—Ghana, Nigeria, Democratic Republic of Congo, South Africa, Kenya, Madagascar and the South Indian Ocean Islands (circled in grey). The numbers next to species names indicate individuals tested. Species with an asterisk indicate a referral to Table S1 for further details regarding taxonomic changes. All species for whom less than 20 individuals were tested are grouped together for visual simplicity with additional details in Table S1.

Rabies is recognized as an underreported disease with a diverse group of viral species as causative agents. Africa is host to diverse lyssaviruses, with surveillance for RABV already poor and even more so for rabies-related lyssaviruses [2,6]. Diagnostic methods, such as the fluorescent antibody test (FAT), where available, cannot distinguish between different lyssaviruses, resulting mostly in the reporting of rabies-positive samples in general with no further characterization of the causative agent [32]. Active surveillance (i.e., the monitoring of bat populations for the presence of lyssaviruses) in wildlife and specifically bats is lacking, also complicated by the neurological pathway of the disease. After infection, the virus enters the neuronal system where it is protected from the immune system, and then moves slowly towards the brain, where it replicates in high titre. Only after this does the virus disseminate to other organs, including intermittent viral shedding in saliva. Lyssaviruses are non-viraemic (absent in the blood) and not excreted in urine or faecal material. Therefore, brain material is the most reliable sample for virus detection, and positive detection is associated with clinical signs. Bats are considered the primary hosts for lyssaviruses [5,6], with 13 extant families and an estimated 58 genera and 339 species reported in Africa [23,35], representing more than 20% of global bat diversity. Very few studies on lyssavirus surveillance for nucleic acid detection in Africa (Table S1) have been published. Subsequently, very few bat genera have been investigated sufficiently (Table 2) compared with species naturally infected with LBV. However, surveillance for lyssaviruses in healthy bat populations using destructive sampling methodologies (since brain material is tested, bats need to be humanely sacrificed) is not very efficient for detecting infections [22]. Over 5000 individual bats have been tested in Africa with a detection rate of less than 1% (Table 2). Bat species known to be reservoirs for lyssaviruses have been disproportionately tested (Figure 2), such as *E. helvum* and *R. aegyptiacus*, compared to other bat species occurring in Africa. Therefore, conclusions regarding the host range, distribution, spillover incidence and spillover hosts of LBV are tentative.

**Figure 3:**
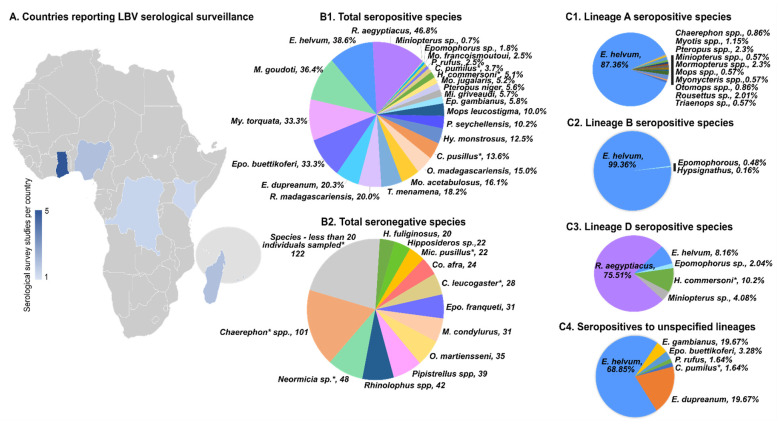
Map depicts the countries where Lagos bat virus serological studies have been reported from bat species, indicated according to the number of studies per country (Ghana, Nigeria, Democratic Republic of Congo, Kenya, Madagascar and Southwestern Indian Islands). Southwestern Indian Ocean islands include Anjouan, La Réunion, Mahé, Mayotte and Mauritius. (**B1**) Bat species from all studies that were seropositive, indicated as percentage positive per species. (**B2**) Bat species from all studies that were seronegative, indicated as number of bats tested per species. Bat species with seropositivity to LBV lineage A (**C1**), lineage B (**C2**), lineage D (**C3**) and unspecified lineages (**C4**) are also indicated as a percentage of the total individuals seropositive for each respective lineage. Table S2 provides more details of taxonomic changes for species indicated with an asterisk and specific sample numbers.

Another approach to investigating lyssaviruses is to conduct serological surveillance. Antibodies may only develop very late in the disease. In particular, bats seem to develop high antibody titres without developing disease, probably due to abortive infection. Therefore, high seroprevalence has been described to be associated with the absence of virus. Serological surveillance usually utilizes non-destructive sampling, i.e., bats are caught, blood is collected and bats are released. For lyssaviruses, the rapid fluorescent focus inhibition test (RFFIT) and the fluorescent antibody virus neutralization test (FAVN) are most frequently used [22,36]. Both methods are based on testing dilutions of sera against a constant challenge virus dose in vitro to quantify virus neutralizing antibodies (VNAs). The presence of VNAs implies previous exposure to the antigen used in the test [37] and is not indicative of an active infection. Like surveillance for nucleic acids, serological surveillance for lyssaviruses has been limited to only a few countries, with members of the Pteropodidae family being disproportionately tested (Table S2 and Table 3). The presence of VNAs has been detected in over 25% of the bats sampled (Table S2, Figure 3), significantly higher than nucleic acid detection rates.

However, the interpretation of serological results is significantly influenced by the cross-reactivity of antibodies to different lyssavirus species and the cut-off thresholds used in the assay [37]. For lyssaviruses, cross-reactivity has been described for viruses belonging to the same phylogroup, such as European bat lyssavirus 1 and Duvenhage virus [38], and for MOKV and LBV [8,10,39]. An attempt to quantify this level of cross-reactivity indicated that only 67% of the antigenic variation was predictable from sequencing information [40]. The interpretation of serological cross-reactivity is further complicated by variable individual responses to exposure with reports that very high antibody titres may enable the neutralization of divergent viruses [41]. Another consideration is the choice of challenge virus used in the test, as cross-reaction was not observed between some LBV isolates. Sera from *E. helvum* in Ghana could neutralize a lineage A virus (isolate 31,225, *E. helvum*, Ghana, 2013) but could not neutralize a lineage B virus (*E. helvum*, Nigeria, 1956) [11].

The antibody prevalence in a specific population is determined by comparing the results obtained to a reference cut-off value [37]. Therefore, the cut-off value would determine if a given sample is positive or negative for antibodies. Studies have reported using different cut-off values in addition to using different assays. Considering the variation that can occur within assays and between assays and laboratories with the lack of an international reference standard for rabies-related viruses, it becomes difficult to compare seroprevalence studies directly. For example, cut-off values used for LBV seroprevalence studies ranged from 1:9 [42] up to 1:40 [43]. This variation in cut-off values directly impacts the assay sensitivity and specificity [37] and, therefore, has a significant effect on data interpretation and ultimately the estimated antibody prevalence. Although serological surveillance is informative, it should be interpreted with caution due to the inherent limitations.

## 5. Lagos Bat Virus Diversity

**Figure 4:**
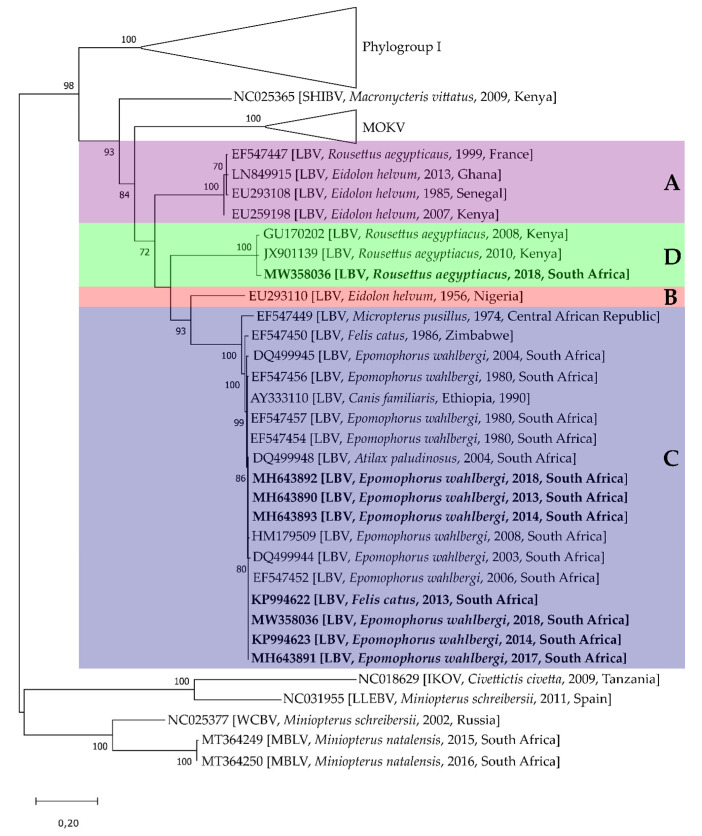
Maximum likelihood phylogenetic tree of the complete nucleoprotein gene of available Lagos bat virus sequences and representatives of all lyssavirus species (Table S3) using the general time reversible model with gamma distribution and invariant sites (GTR + G+I). Lagos bat virus lineages (**A**–**D**) are indicated in capital letters. The reliability of the branching pattern was statistically evaluated by bootstrap analysis of 1000 replications and are indicated at the nodes. Scale bar indicates the number of substitutions per site. Sequences generated in this report are indicated in bold.

With the description of several viruses related to RABV during the mid-twentieth century, the *Lyssavirus* genus was initially divided into four serotypes, i.e., RABV (serogroup 1), LBV (serogroup 2), MOKV (serogroup 3) and DUVV (serogroup 4), based on reaction patterns with monoclonal antibodies [44]. Additional isolations of lyssaviruses followed and, together with improvements in molecular techniques for genetic characterization, led to the description of lyssavirus genotypes that followed the serogroup designation with an additional two genotypes [45]. During the 1990s, it was suggested that lyssavirus genotypes are divided based on a threshold nucleotide identity of 80% and 93% for amino acids [45,46]. The first comprehensive phylogenetic studies of LBV indicated that these viruses could be divided into three distinct lineages, i.e., lineage A–C. Viruses belonging to lineage A were also shown to exceed the genotype threshold compared to lineages B and C, suggesting that lineage A should constitute a new genotype [16,47]. It was also during this time that the ICTV approved the classification of lyssaviruses into species. This complex taxonomic entity would, therefore, consider several characteristics and not rely solely on genetic distance. Although LBV isolates exceed current genetic species demarcation criteria, it is not regarded as separate species [3]. An additional lineage D was also described from Kenya and more recently from South Africa (this report) with *R. aegyptiacus* as the only known associated host (Figure 4). The first full genome for LBV was only published in 2008, and currently, there are seven full genomes available with the first and only lineage C LBV genome (this report) available for comparison. The genetic identity for complete concatenated genomes ranged from 76% to 98.8% for nucleotides and 87.8% to 99.7% for amino acids (Table 4). It appears that LBV displays genetic spatiotemporal stability. For lineage A viruses, the only lineage with multiple full genomes available, that were detected 28 years apart from West and East Africa, less than 1.5% nucleotide divergence is observed. This suggests that the viruses of each lineage are adapted to specific hosts [10]. Due to limited surveillance in Africa, no conclusive assumptions can be made regarding the host range of LBV; however, available data suggest that lineage A and B viruses are associated with *E. helvum*, lineage C with *E. wahlbergi* and lineage D with *R. aegytiacus*.

## 6. Pathogenesis

Since LBV was shown to be serologically related to RABV [8], the question arose on whether pathogenicity, disease development and pathological findings are comparable. The first study investigating the pathogenicity (i.e., the ability to cause disease) of LBV was performed in monkeys and dogs [48]. Results indicated that similar to RABV, a preference for the central nervous system was observed. However, reduced pathogenicity was observed when these animals were infected via the intramuscular route (IM). Several decades after this, a comparative study investigating the differences in pathogenicity, immunogenicity and genetics of lyssaviruses was performed [39]. Results indicated that phylogroup II viruses (i.e., LBV and MOKV) were not pathogenic via the IM route. This was due to an amino acid substitution in the glycoprotein gene (Arginine at position 333). Considering the increased genetic diversity described for LBV, the decreased peripheral pathogenicity was revisited in subsequent investigations with results indicating the equal, and in some cases, increased pathogenicity of LBV compared to RABV [17,49]. These studies indicated that a lyssavirus species’ pathogenicity should not be based on a single isolate and that a single amino acid change cannot predict pathogenicity. Several domains considered to be important for pathogenicity are conserved in LBV isolates; however, several other mutations also occur that reportedly result in reduced pathogenicity (Table 5). Based on genetic data and experimental infections in mice, it would appear that there is some cooperativity between pathogenic domains within LBV but that currently unknown additional domains or factors exist that contribute to the pathogenicity of lyssaviruses [17].

**Figure 5:**
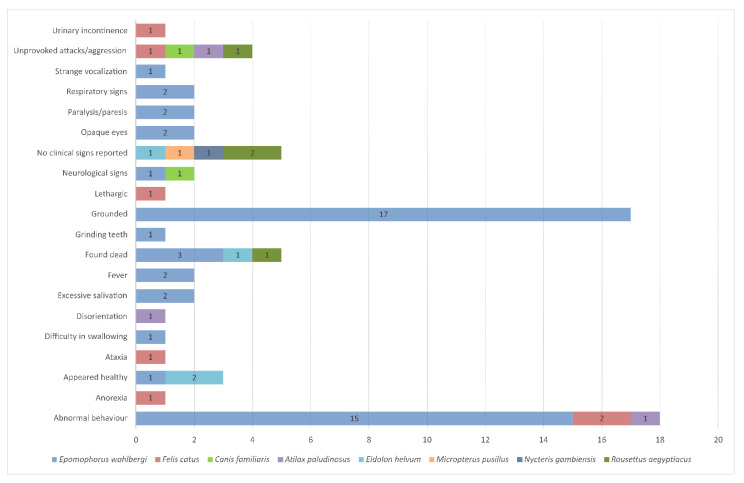
Reported clinical signs for animals naturally infected with Lagos bat virus.

Experimental infections (Table 6) have mostly been performed in non-reservoir hosts, such as mice, potentially resulting in inaccurate disease progression assumptions in naturally infected animals. More recently, the pathogenesis of LBV in one of its natural hosts, i.e., *E. helvum,* was investigated [63,64]. Similar mortality rates, as observed in other animals, were reported (Table 6). Clinical signs observed in experimentally infected bats appear similar to animals naturally infected with LBV (Figure 5). Additionally, lyssavirus antigens were detected in tongue epithelium and, together with the salivary glands, are probable virus excretion sites [63,64]. However, it was noted that for four bats, experimentally infected with different LBV doses (10^0.1^–10^4.1^ TCID_50_), no clinical signs were observed before the bats were found dead [63]. This phenomenon has also been reported in bats naturally infected with LBV and warrants further investigation [1,11].

The mechanisms of virus maintenance and transmission of lyssaviruses in bats are not well understood and could be influenced by various factors [2,26]. The experimental infection of *E. helvum* with LBV indicated that bats that survived the challenge had developed virus neutralizing antibodies (53%), suggesting that bats can develop an immune response without clinical disease development and thus not excrete virus [63]. This would support surveillance results, indicating high seroprevalence and low virus detection in Africa.

## 7. Prevention

Commercial rabies biologics (rabies immunoglobulin (RIG) and vaccines) are based on RABV [65], and it is essential to assess their efficacy against other lyssaviruses, including LBV. Protection against disease development following exposure is reliant on both the innate (i.e., the basic immune system that induces non-specific resistance to disease) and the adaptive immune responses (i.e., highly specialized and systemic process). The presence of VNAs has been shown to be essential for protection against a productive infection [63,66]. Initial neutralization tests in mice indicated that limited cross-reactivity occurs for LBV and that rabies vaccines might not confer protection [7,8]. This conclusion was further supported by additional studies showing that sera obtained from rabies-vaccinated people demonstrated significantly less neutralizing activity against LBV compared to viruses of phylogroup I [41,67]. This lack of protection against LBV has also been noted in a challenge study performed in mice [68] in addition to LBV infections reported in previously vaccinated domestic animals [15,27,28]. The titre of VNAs above a certain threshold is usually interpreted as indicative of protective immunity. Based on current data, the VNAs produced following rabies vaccines will likely not be protective against LBV infection. However, it has been suggested that several other components of the immune response (that is currently not completely understood or characterized) may play an important role in protection in addition to VNAs [69]. No human LBV cases have been reported to date; however, there is a potential spillover risk, since several human exposures have been associated with animals infected with LBV. Human rabies in Africa is also underestimated, frequently misdiagnosed and has limited diagnostic capacity on the continent, not often typed or molecularly characterized [70,71,72,73], and may simply have been missed. Additional treatment options, such as updated vaccines and alternatives to rabies immunoglobulin, should target a broader diversity of lyssaviruses [74,75]. Several strategies and potential pan-lyssavirus vaccines have been formulated. However, due to some inherent limitations (such as safety concerns, associated costs, inadequate immune responses and efficacy), a suitable alternative to the current rabies vaccines have not been established [74,76,77,78,79]. Public and veterinary health risks are suggested to be low, as demonstrated by the prevalence of infections in animals and humans. Since comprehensive data on the cross-reactivity of current biologicals are lacking, the cross-protection afforded by rabies biologicals is not known. As such, all bat exposures should be prioritized as category III exposures, as recommended by the World Health Organization (WHO), with post-exposure prophylaxis consisting of extensive and thorough washing of the wound, rabies immunoglobulin and vaccine administration [65].

## 8. Conclusions

Lagos bat virus is a unique endemic African lyssavirus, and detections remain rare and sporadic, mostly linked to where specific surveillance programs have been implemented. Surveillance efforts usually include only limited species diversity, mainly focused on those species previously implicated in infection. Viral nucleic acid detection has indicated an association of LBV with specific bat species, predominantly frugivorous bats. LBV lineages also appear to be associated with particular bat species. Serological surveillance has implicated a much broader geographical and species range, highlighting the lack of surveillance. Lyssaviruses are neurotropic and only disseminate late in infection to other organs and potential excretions; however, virus shedding may be intermittent. As such, the most reliable sample for viral nucleic acid detection will be post-mortem brain material. Detection rates using this strategy are very low (below 1%). Considering the vital part bats play in ecosystems, combined with reduced population sizes and increased habitat destruction [26,80], this suggests that a re-evaluation of surveillance initiatives is needed. Surveillance efforts to detect LBV (and other lyssaviruses) should not be focused on killing apparently healthy animals. It should instead target animals displaying abnormal behaviour or clinical signs suggestive of rabies.

Serology on serum collected from healthy bat populations can be used as a surveillance tool through non-destructive sampling to obtain an estimate of the circulating diversity in specific species in a geographical space. However, serological studies’ inherent limitations, including the potential cross-reactivity of lyssaviruses, should be considered. Seroprevalence in natural bat populations is significantly higher than the rate of virus detection. Specific bat species are regarded as reservoirs of lyssavirus infection and can generate an antibody response after initial infection. This mechanism is still not understood and potentially attributed to an abortive infection outside the central nervous system [81]. Spillover infections of LBV have been reported in various animals, several that have been previously vaccinated against rabies. Although no human LBV infections have been reported, such a spillover infection is possible with several exposure events being described. Such spillover infections usually result in dead-end events with no onward transmission [82,83]. Several factors have been hypothesized to play a role in onward transmission, including the incubation period, virus titre present in saliva, inherent features of the viral lineage involved, virus–host interactions and phylogenetic distance between the reservoir and non-reservoir host [83,84]. Although our understanding of the influence and impact of these factors on virus transmission remain limited, it appears that shorter incubation periods and reduced viral titre in the salivary glands of non-reservoir hosts restrict onward transmission [84].

There is currently a lack of knowledge on the host–pathogen interactions at the level of the pathogen (i.e., LBV) and the host (i.e., bats). Our understanding of the LBV infection dynamics, factors and mechanisms necessary for LBV transmission and maintenance, spillover mechanisms and information regarding bat population dynamics remains incomplete. Only once surveillance efforts are aligned with bat ecology and conservation using a one-health approach can we begin to address these fundamental but inherently complicated questions.

## Figures and Tables

**Table 1:** Lyssavirus classification, commonly associated hosts and geographical distribution.

Lyssavirus Species	Phylogroup	Geographical Distribution	Host(s) ^1^
*Aravan lyssavirus*	I	Eurasia	*Myotis blythi*
*Australian bat lyssavirus*	I	Australasia	*Pteropus alecto* *Saccolaimus flaviventris*
*Bokeloh bat lyssavirus*	I	Europe	*Myotis nattereri*
*Duvenhage lyssavirus*	I	Africa	*Nycteris thebaica*
*European bat 1 lyssavirus*	I	Europe	*Eptesicus serotinus*
*European bat 2 lyssavirus*	I	Europe	*Myotis daubentonii*
*Gannoruwa bat lyssavirus*	I	Asia	*Pteropus medius*
*Ikoma lyssavirus*		Africa	*Civettictis civetta* ^2^
*Irkut lyssavirus*	I	Eurasia	*Murina leucogaster*
*Khujand lyssavirus*	I	Eurasia	*Myotis mystacinus*
*Kotalathi bat lyssavirus* ^3^	I	Europe	*Myotis brandtii*
*Lagos bat lyssavirus*	II	Africa	*Eidolon helvum* *Rousettus aegyptiacus* *Epomophorus wahlbergi*
*Lleida bat lyssavirus*		Europe	*Miniopterus schreibersii*
*Matlo bat lyssavirus* ^4^		Africa	*Miniopterus natalensis*
*Mokola lyssavirus*	II	Africa	*Felis catus* ^2^
*Rabies lyssavirus*	I	Almost worldwide	*Most mammalian species*
*Shimoni bat lyssavirus*	II	Africa	*Macronycteris vittatus*
*Taiwan bat lyssavirus*	I	Asia	*Pipistrellus abramus*
*West Caucasian bat lyssavirus*		Eurasia	*Miniopterus schreibersii*

^1^ Only the most frequently reported species are indicated; ^2^ unknown reservoir host, listed species considered spillover hosts. ^3^ Tentative species; ^4^ potential novel species.

**Table 2:** Summary of bat families and genera tested during surveillance for lyssavirus nucleic acid in Africa (details of studies can be found in Table S1).

Host Family	Genera	Species ^1^	Genera (Tested Species ^2^)	Tested Individuals	Positive Individuals	Virus
PTEROPODIDAE	13	44	5 (8)	2350	4	Lagos bat virus
HIPPOSIDERIDAE	4	21	2 (3)	62	1	Shimoni bat virus
MEGADERMATIDAE	2	2	2 (2)	29	0	
RHINOLOPHIDAE	1	38	1 (11)	321	0	
RHINONYCTERIDAE	3	6	2 (2)	21	0	
RHINOPOMATIDAE	1	3	1 (1)	4	0	
MYZOPODIDAE	1	2	0 (0)	0	0	
EMBALLONURIDAE	4	12	2 (3)	44	0	
NYCTERIDAE	1	15	1 (3)	69	1	Duvenhage virus
MOLOSSIDAE	7	44	4 (7)	333	0	
CISTUGONIDAE	1	2	0 (0)	0	0	
MINIOPTERIDAE	1	26	1 (5)	555	2	Matlo bat lyssavirus
VESPERTILIONIDAE	19	124	11 (19)	303	0	
Not determined	-	-	-	1193	0	
TOTALS	58	339	32 (64)	5284	8	0.15%

^1^ Total species counts are fluid due to ongoing species rearrangement or reassignment; ^2^ not all bats sampled are identified at species level.

**Table 3:** Summary of bat families and genera tested during surveillance for Lagos bat virus neutralizing antibodies in Africa (details of studies can be found in Table S2).

Host Family	Genera	Species ^1^	Genera (Tested Species ^2^)	Tested Individuals	Positive Individuals	Percentage Positive
PTEROPODIDAE	13	44	8 (14)	3064	1048	34.2
HIPPOSIDERIDAE	4	21	2 (3)	148	5	3.4
MEGADERMATIDAE	2	2	1 (1)	3	0	0
RHINOLOPHIDAE	1	38	1 (1)	44	0	0
RHINONYCTERIDAE	3	6	1 (2)	19	2	17.8
RHINOPOMATIDAE	1	3	0 (0)	0	0	0
MYZOPODIDAE	1	2	0 (0)	0	0	0
EMBALLONURIDAE	4	12	1 (1)	24	0	0
NYCTERIDAE	1	15	1 (1)	14	0	0
MOLOSSIDAE	7	44	3 (13)	517	20	3.9
CISTUGONIDAE	1	2	0 (0)	0	0	0
MINIOPTERIDAE	1	26	1 (5)	343	4	1.2
VESPERTILIONIDAE	19	124	8 (8)	120	4	3.3
TOTALS	58	339	27 (49)	4296	1083	25.2

^1^ Total species counts are fluid due to ongoing species rearrangement or reassignment; ^2^ not all bats sampled are identified at species level.

**Table 4:** Nucleotide (grey) and amino acid identity of concatenated Lagos bat virus genomes.

	Lineage A			Lineage B	Lineage C	Lineage D	
	LN849915 ^1^	NC020807 ^2^	EU259198 ^3^	EU293110 ^4^	MH643893 ^5^	GU170202 ^6^	JX901139 ^7^
LN849915		98.7	98.8	76.3	76	76.3	76.4
NC020807	99.7		98.8	76.2	76	76.2	76.4
EU259198	99.6	99.6		76.2	76	76.3	76.5
EU293110	87.9	87.9	87.8		79.6	76.7	76.9
MH643893	88.9	88.9	88.8	92.2		77.1	77.2
GU170202	89.5	89.6	89.5	88.4	89.2		98.3
JX901139	89.5	89.6	89.5	88.3	89.3	99.4	

^1^*E. helvum*, 2013, Ghana; ^2^*E. helvum*, 1985, Senegal; ^3^*E. helvum*, 2007, Kenya; ^4^*E. helvum*, 1956, Nigeria; ^5^*E. wahlbergi*, 2014, South Africa; ^6^*R. aegyptiacus*, 2008, Kenya; ^7^*R. aegyptiacus*, 2010, Kenya.

**Table 5:** Conservation of amino acids ^1^ of pathogenic domains on Lagos bat virus genomes.

Protein ^2^	Region	Ref ^3^	Lineage A			Lineage B	Lineage C	Lineage D	
			EU 259198	NC 020807	LN 849915	EU 293110	MH 643893	JX 901139	GU 170202
N	273	[50]	F	F	F	F	F	F	F
	394		F	F	F	F	F	F	F
P	144–148	[51]	RQTQT	RQTQT	RQTQT	KQTQT	KQTQT	KNTQT	KNTQT
M	22–25	[52]	ASAP	ASAP	ASAP	PSAP	PSAP	ASAP	ASAP
	35–38	[53]	PPEY	PPEY	PPEY	PPEY	PPEY	PPEY	PPEY
	77	[54]	K	K	K	K	K	K	K
	81		N	N	N	N	S	N	N
	95	[55]	I	I	I	M	V	I	I
G	132	[56]	L	L	L	L	L	L	L
	194	[57]	T	T	T	T	T	T	T
	198	[56]	R	R	R	K	K	R	R
	242	[58]	S	S	S	S	S	S	S
	255		D	D	D	N	N	N	N
	268		I	I	I	V	I	L	L
	318	[59]	L	L	L	L	I	L	L
	352		M	M	M	V	V	L	L
	330–333	[60,61,62]	KRVD	KRVD	KRVD	LKVD	LRVD	RRVD	RRVD

^1^ Amino acid abbreviations. A: Alanine; E: Glutamic acid; F: Phenylalanine; I: Isoleucine; K: Lysine; L: Leucine; M: Methionine; N: Asparagine; P: Proline; Q: Glutamine; R: Arginine; S: Serine; T: Threonine; V: Valine; Y: Tyrosine; ^2^ Gene abbreviations. N: nucleoprotein gene; P: phosphoprotein gene; M: matrix protein gene; G: glycoprotein gene; ^3^ reference for the description and the influence of the mutation.

**Table 6:** Experimental infections of animals with Lagos bat virus.

Host	Route1	Dose	Isolate	Clinical Signs	Mortality	Reference
*Macaca mulatta*	IC	6.2 log ICDL_50_/mL	8619NGA (EU293110)	Agitation	100% (*n* = 2)	[48]
	IM			Paresis	20% (*n* = 5)	
Canine	IC			Depression, incoordination	100% (*n* = 2)	
	IM			No clinical signs	0% (*n* = 2)	
Mice (BALB/3H)	IM	3 × 10^5^ LD_50_	8619NGA (EU293110)	N/A	0%	[39]
Mice (ICR)	IC	10^2^ MICLD_50_	0406SEN (EU93108)	Not specified	100% (*n* = 5)	[49]
	IM	10^3^ MICLD_50_			60% (*n* = 5)	
	IM	10^6^ MICLD_50_			100% (*n* = 5)	
	IC	10^2^ MICLD_50_	Afr1999 (EF547447)		100% (*n* = 5)	
	IM	10^3^ MICLD_50_			20% (*n* = 5)	
	IM	10^6^ MICLD_50_			100% (*n* = 5)	
	IC	10^2^ MICLD_50_	Zim1986 (EF547450)		100% (*n* = 5)	
	IM	10^3^ MICLD_50_			20% (*n* = 5)	
	IM	10^6^ MICLD_50_			20% (*n* = 5)	
	IM	10^2^ MICLD_50_	CAR1974 (EF547449)		100% (*n* = 5)	
	IM	10^3^ MICLD_50_			0% (*n* = 5)	
	IM	10^6^ MICLD_50_			20% (*n* = 5)	
	IC	10^2^ MICLD_50_	Mong2004 (DQ499948)		100% (*n* = 5)	
	IM	10^3^ MICDL_50_			20% (*n* = 5)	
	IM	10^6^ MICLD_50_			20% (*n* = 5)	
	IC	10^2^ MICLD_50_	SA2004 (EF547458)		100% (*n* = 5)	
	IM	10^3^ MICLD_50_			40% (*n* = 5)	
	IM	10^6^ MICLD_50_			60% (*n* = 5)	
	IC	10^2^ MICLD_50_	SA2003 (EF547421)		100% (*n* = 5)	
	IM	10^3^ MICLD_50_			20% (*n* = 5)	
	IM	10^6^ MICLD_50_			20% (*n* = 5)	
	IC	10^2^ MICLD_50_	SA2006 (EF547422)		100% (*n* = 5)	
	IM	10^3^ MICLD_50_			0% (*n* = 5)	
	IM	10^6^ MICLD_50_			20% (*n* = 5)	
Mice (BALB/c)	IM	10^3^ TCID_50_	SA2008 (HM179509)	Hind limb paralysis, ruffled fur, weight loss, walking in circles	0% (*n* = 4)	[17]
		10^5^ TCID_50_	SA2008 (HM179509)		50% (*n* = 4)	
			8619NGA (EU293110)		0% (*n* = 4)	
		10^7^ TCID_50_	SA2004 (EF547458)		60% (*n* = 5)	
		10^8^ TCID_50_	Mong2004 (DQ499948)		40% (*n* = 5)	
		10^8^ TCID_50_	Afr1999 (EF547447)		80% (*n* = 5)	
*Eidolon helvum*	IC	10^3.5^ TCID_50_	0406SEN (EU293108)	Hindleg paresis, muscle spasms, hyperaesthesia, foam around mouth, anorexia	100% (*n* = 3)	[64]
			8619NGA (EU293110)		100% (*n* = 3)	
			31225 (LN849915)		100% (*n* = 3)	
*Eidolon helvum*	IM	10^0.1^ TCID_50_	31225 (LN849915)	No clinical signs	25% (*n* = 4)	[63]
		10^1.1^ TCID_50_		Vocalization, muscle spasms, salivation, aggression	50% (*n* = 4)	
		10^2.1^ TCID_50_			100% (*n* = 4)	
		10^3.1^ TCID_50_			50% (*n* = 4)	
		10^4.1^ TCID_50_			50% (*n* = 4)	

## Data Availability

Data presented in this study is openly available at ncbi.nlm.nih.gov (accessed on 25 February 2021) and the supplementary material (Tables S1–S3).

## Supplementary Materials

The following are available online at https://www.mdpi.com/article/10.3390/v13040576/s1, Table S1: Details of surveillance studies for lyssavirus nucleic acid detection in Africa; Table S2. Details of serological surveillance studies for Lagos bat virus in Africa; Table S3. Details of lyssavirus sequences used for phylogenetic analysis.
