# Supplementary material for: Lagos Bat Virus, an Under-Reported Rabies-Related Lyssavirus
Source: Viruses. 2021 Mar 29;13(4):576. doi: 10.3390/v13040576 (PMC8067007; doi:10.3390/v13040576)
Supplement: Supplementary file 1 [file viruses-13-00576-s001.zip › Table S1. Details of surveillance studies for lyssavirus nucleic acid detection in Africa.docx]

Table S1. Details of surveillance studies for lyssavirus nucleic acid detection in Africa

| Bat species | Proposed taxonomic update^1^ | Family | Number tested | Number positive | Country | Lyssavirus detected | Reference |
| --- | --- | --- | --- | --- | --- | --- | --- |
| *Eidolon helvum* |  | PTEROPIDAE | 42 | 1 | Nigeria | Lagos bat virus | [1] |
| *Chaerephon pumilus* | *Mops pumilus* | MOLOSSIDAE | 59 | 0 | Madagascar |  | [2] |
| *Scotophilus dinganii* |  | VESPERTILLIONIDAE | 3 | 0 | South Africa |  | [3] |
| *Pipistrellus nanus* | *Laephotis nanus, Afronycteris nana* | VESPERTILLIONIDAE | 6 | 0 | South Africa |  |  |
| *Epomophorus wahlbergi* |  | PTEROPIDAE | 29 | 0 | South Africa |  |  |
| *Chaerephon pumilus* | *Mops pumilus* | MOLOSSIDAE | 6 | 0 | South Africa |  |  |
| *Rhinolophus* sp*.* |  | RHINOLOPHIDAE | 12 | 0 | South Africa |  |  |
| *Miniopterus natalensis* |  | MINIOPTERIDAE | 10 | 0 | South Africa |  |  |
| *Nycteris thebaica* |  | NYCTERIDAE | 3 | 0 | South Africa |  |  |
| *Otomops martiensseni* |  | MOLOSSIDAE | 5 | 0 | South Africa |  |  |
| Not determined |  | Not determined | 6 | 0 | South Africa |  |  |
| *Pipistrellus hesperidus* |  | VESPERTILLIONIDAE | 3 | 0 | South Africa |  |  |
| *Rhinolophus darlingi* |  | RHINOLOPHIDAE | 1 | 0 | South Africa |  |  |
| *Pipistrellus kuhli* | *Pipistrellus hesperidus* | VESPERTILLIONIDAE | 2 | 0 | South Africa |  |  |
| *Hipposideros caffer* |  | HIPPOSIDERIDAE | 2 | 0 | South Africa |  |  |
| *Rhinolophus hildebrandti* |  | RHINOLOPHIDAE | 1 | 0 | South Africa |  |  |
| *Neoromicia africana* | *Laephotis* sp*.* | VESPERTILLIONIDAE | 1 | 0 | South Africa |  |  |
| *Rhinolophus clivosus* |  | RHINOLOPHIDAE | 7 | 0 | South Africa |  |  |
| *Neoromicia capensis* | *Laephotis capensis* | VESPERTILLIONIDAE | 2 | 0 | South Africa |  |  |
| *Miniopterus* sp. |  | MINIOPTERIDAE | 202 | 0 | Kenya |  | [4] |
| *Rhinolophus* sp. |  | RHINOLOPHIDAE | 61 | 0 | Kenya |  |  |
| *Rousettus aegyptiacus* |  | PTEROPIDAE | 345 | 0 | Kenya |  |  |
| *Hipposideros ruber* |  | HIPPOSIDERIDAE | 6 | 0 | Kenya |  |  |
| *Chaerephon pumilus* | *Mops pumilus* | MOLOSSIDAE | 24 | 0 | Kenya |  |  |
| *Eidolon helvum* |  | PTEROPIDAE | 109 | 1 | Kenya | Lagos bat virus |  |
| *Epomophorus labiatus* |  | PTEROPIDAE | 7 | 0 | Kenya |  |  |
| *Chaerephon* sp*.* | *Mops* sp. | MOLOSSIDAE | 51 | 0 | Kenya |  |  |
| *Rousettus angolensis* |  | PTEROPIDAE | 10 | 0 | Kenya |  |  |
| *Miniopterus inflatus* |  | MINIOPTERIDAE | 12 | 0 | Kenya |  |  |
| *Eptesicus tenuipinnis* | *Laephotis tenuipinnis* | VESPERTILLIONIDAE | 4 | 0 | Kenya |  |  |
| *Rhinolophus landeri* |  | RHINOLOPHIDAE | 18 | 0 | Kenya |  |  |
| *Otomops martiensseni* |  | MOLOSSIDAE | 19 | 0 | Kenya |  |  |
| *Pipistrellus* sp. |  | VESPERTILLIONIDAE | 5 | 0 | Kenya |  |  |
| *Epomophorus wahlbergi* |  | PTEROPIDAE | 12 | 0 | Kenya |  |  |
| *Miniopterus africanus* |  | MINIOPTERIDAE | 29 | 0 | Kenya |  |  |
| *Rhinolophus hildebrandti* |  | RHINOLOPHIDAE | 22 | 0 | Kenya |  |  |
| *Nycteris sp.* |  | NYCTERIDAE | 2 | 0 | Kenya |  |  |
| *Neoromicia* sp. | *Laephotis* sp. | VESPERTILLIONIDAE | 2 | 0 | Kenya |  |  |
| *Coleura afra* |  | EMBALLONURIDAE | 36 | 0 | Kenya |  |  |
| *Taphozous* sp. |  | EMBALLONURIDAE | 2 | 0 | Kenya |  |  |
| *Carioderma cor* |  | MEGADERMITIDAE | 26 | 0 | Kenya |  |  |
| Not determined |  | Not determined | 4 | 0 | Kenya |  |  |
| *Hipposideros commersoni* | *Macronycteris vittatus* | HIPPOSIDERIDAE | 16 | 0 | Kenya |  |  |
| *Miniopterus minor* |  | MINIOPTERIDAE | 134 | 0 | Kenya |  |  |
| *Nycteris hispida* |  | NYCTERIDAE | 4 | 0 | Kenya |  |  |
| *Triaenops persicus* | *Triaenops afer* | RHINONYCTERIDAE | 16 | 0 | Kenya |  |  |
| *Taphozous hildegardeae* |  | EMBALLONURIDAE | 3 | 0 | Kenya |  |  |
| *Scotophilus* sp*.* |  | VESPERTILLIONIDAE | 1 | 0 | Kenya |  |  |
| Not determined |  | Not determined | 616 | 1 | Kenya | Shimoni bat virus | [5] |
| *Eidolon helvum* |  | PTEROPIDAE | 223 | 0 | Nigeria |  | [6] |
| *Micropterus pusillus* | *Epomophorus pusillus* | PTEROPIDAE | 55 | 0 | Nigeria |  |  |
| *Epomophorus gambianus* |  | PTEROPIDAE | 12 | 0 | Nigeria |  |  |
| Not determined |  | Not determined | 17 | 0 | Nigeria |  |  |
| *Rhinolophus alcyone* |  | RHINOLOPHIDAE | 43 | 0 | Nigeria |  |  |
| *Eidolon helvum* |  | PTEROPIDAE | 122 | 0 | Ghana |  | [7] |
| *Chaerephon pumilus* | *Mops pumilus* | MOLOSSIDAE | 33 | 0 | Nigeria |  | [8] |
| *Eidolon helvum* |  | PTEROPIDAE | 244 | 0 | Nigeria |  |  |
| *Epomophorus franquetti* | *Epomops franqueti* | PTEROPIDAE | 12 | 0 | Nigeria |  |  |
| *Epomophorus gambianus* |  | PTEROPIDAE | 5 | 0 | Nigeria |  |  |
| *Lavia frons* |  | MEGADERMITIDAE | 3 | 0 | Nigeria |  |  |
| *Nycteris macrotis* |  | NYCTERIDAE | 18 | 0 | Nigeria |  |  |
| *Rhinolophus landeri* |  | RHINOLOPHIDAE | 37 | 0 | Nigeria |  |  |
| *Rhinopoma microphyllum* |  | RHINOPOMATIDAE | 4 | 0 | Nigeria |  |  |
| *Eidolon helvum* |  | PTEROPIDAE | 630 | 1 | Ghana | Lagos bat virus | [9] |
| Not determined |  | Not determined | 550 | 0 | Southwest Indian Ocean Islands |  | [10] |
| *Eidolon helvum* |  | PTEROPIDAE | 19 | 0 | Democratic Republic of Congo |  | [11] |
| *Micropteropus pussilus* | *Epomophorus pussilus* | PTEROPIDAE | 1 | 0 | Democratic Republic of Congo |  |  |
| *Chaerephon pumilus* | *Mops pumilus* | MOLOSSIDAE | 24 | 0 | Democratic Republic of Congo |  |  |
| *Chaerephon sp.* | *Mops sp.* | MOLOSSIDAE | 22 | 0 | Democratic Republic of Congo |  |  |
| *Glauconycteris argentata* |  | VESPERTILLIONIDAE | 1 | 0 | Democratic Republic of Congo |  |  |
| *Hipposideros fuliginosus* |  | HIPPOSIDERIDAE | 20 | 0 | Democratic Republic of Congo |  |  |
| *Hipposideros gigas* | *Macronycteris gigas* | HIPPOSIDERIDAE | 1 | 0 | Democratic Republic of Congo |  |  |
| *Hypsignathus monstrosus* |  | PTEROPIDAE | 2 | 0 | Democratic Republic of Congo |  |  |
| *Megaglossus woermanni* |  | PTEROPIDAE | 8 | 0 | Democratic Republic of Congo |  |  |
| *Mimetillus moloneyi* |  | VESPERTILLIONIDAE | 1 | 0 | Democratic Republic of Congo |  |  |
| *Miniopterus sp.* |  | MINIOPTERIDAE | 39 | 0 | Democratic Republic of Congo |  |  |
| *Mops condylurus* |  | MOLOSSIDAE | 33 | 0 | Democratic Republic of Congo |  |  |
| *Myonycteris torquata* |  | PTEROPIDAE | 6 | 0 | Democratic Republic of Congo |  |  |
| *Myotis sp.* |  | VESPERTILLIONIDAE | 1 | 0 | Democratic Republic of Congo |  |  |
| *Neoromicia sp.* |  | VESPERTILLIONIDAE | 1 | 0 | Democratic Republic of Congo |  |  |
| *Pipistrellus sp.* |  | VESPERTILLIONIDAE | 36 | 0 | Democratic Republic of Congo |  |  |
| *Rhinolophus sp.* |  | RHINOLOPHIDAE | 1 | 0 | Democratic Republic of Congo |  |  |
| *Scotophilus dinganii* |  | VESPERTILLIONIDAE | 2 | 0 | Democratic Republic of Congo |  |  |
| *Eidolon helvum* |  | PTEROPIDAE | 46 | 0 | Ghana |  | [12] |
| *Chaerephon ansorgei* | *Mops ansorgei* | MOLOSSIDAE | 2 | 0 | South Africa |  | [13] |
| *Chaerephon pumilus* | *Mops pumilus* | MOLOSSIDAE | 18 | 0 | South Africa |  |  |
| *Cloeotis percivali* |  | RHINONYCTERIDAE | 5 | 0 | South Africa |  |  |
| *Eptesicus hottentotus* |  | VESPERTILLIONIDAE | 1 | 0 | South Africa |  |  |
| *Glauconycteris variegata* |  | VESPERTILLIONIDAE | 4 | 0 | South Africa |  |  |
| *Hipposideros caffer* |  | HIPPOSIDERIDAE | 17 | 0 | South Africa |  |  |
| *Kerivoula argentata* |  | VESPERTILLIONIDAE | 1 | 0 | South Africa |  |  |
| *Laephotis botswanae* |  | VESPERTILLIONIDAE | 2 | 0 | South Africa |  |  |
| *Miniopterus fraterculus* |  | MINIOPTERIDAE | 5 | 0 | South Africa |  |  |
| *Miniopterus natalensis* |  | MINIOPTERIDAE | 124 | 2 | South Africa | Matlo bat lyssavirus |  |
| *Mops condylurus* |  | MOLOSSIDAE | 9 | 0 | South Africa |  |  |
| *Mops midas* |  | MOLOSSIDAE | 3 | 0 | South Africa |  |  |
| *Myotis tricolor* |  | VESPERTILLIONIDAE | 11 | 0 | South Africa |  |  |
| *Myotis welwitschii* |  | VESPERTILLIONIDAE | 1 | 0 | South Africa |  |  |
| *Neoromicia capensis* | *Laephotis capensis* | VESPERTILLIONIDAE | 67 | 0 | South Africa |  |  |
| *Neoromicia cf. helios* | *Laephotis helios* | VESPERTILLIONIDAE | 4 | 0 | South Africa |  |  |
| *Neoromicia nana* | *Laephotis nanus/Afronycteris nana* | VESPERTILLIONIDAE | 13 | 0 | South Africa |  |  |
| *Neoromicia rendalli* | *Laephotis rendalli/Pseudoromicia rendalli* | VESPERTILLIONIDAE | 1 | 0 | South Africa |  |  |
| *Neoromicia zuluensis* | *Laephotis zuluensis* | VESPERTILLIONIDAE | 3 | 0 | South Africa |  |  |
| *Nycteris thebaica* |  | NYCTERIDAE | 42 | 1 | South Africa | Duvenhage virus |  |
| *Nycticeinops schlieffeni* |  | VESPERTILLIONIDAE | 8 | 0 | South Africa |  |  |
| *Otomops martiensseni* |  | MOLOSSIDAE | 7 | 0 | South Africa |  |  |
| *Pipistrellus hesperidus* |  | VESPERTILLIONIDAE | 20 | 0 | South Africa |  |  |
| *Pipistrellus rusticus* |  | VESPERTILLIONIDAE | 35 | 0 | South Africa |  |  |
| *Rhinolophus blasii* |  | RHINOLOPHIDAE | 12 | 0 | South Africa |  |  |
| *Rhinolophus clivosus* |  | RHINOLOPHIDAE | 30 | 0 | South Africa |  |  |
| *Rhinolophus damarensis* |  | RHINOLOPHIDAE | 5 | 0 | South Africa |  |  |
| *Rhinolophus darlingi* |  | RHINOLOPHIDAE | 5 | 0 | South Africa |  |  |
| *Rhinolophus hildebrandtii s.l.* |  | RHINOLOPHIDAE | 3 | 0 | South Africa |  |  |
| *Rhinolophus denti* |  | RHINOLOPHIDAE | 5 | 0 | South Africa |  |  |
| *Rhinolophus landeri* |  | RHINOLOPHIDAE | 1 | 0 | South Africa |  |  |
| *Rhinolophus simulator* |  | RHINOLOPHIDAE | 50 | 0 | South Africa |  |  |
| *Rhinolophus smithersi* |  | RHINOLOPHIDAE | 4 | 0 | South Africa |  |  |
| *Rhinolophus swinnyi* |  | RHINOLOPHIDAE | 3 | 0 | South Africa |  |  |
| *Sauromys petrophilus* |  | MOLOSSIDAE | 1 | 0 | South Africa |  |  |
| *Scotophilus dinganii* |  | VESPERTILLIONIDAE | 49 | 0 | South Africa |  |  |
| *Scotophilus leucogaster* |  | VESPERTILLIONIDAE | 5 | 0 | South Africa |  |  |
| *Scotophilus viridis* |  | VESPERTILLIONIDAE | 7 | 0 | South Africa |  |  |
| *Tadarida aegyptiaca* |  | MOLOSSIDAE | 17 | 0 | South Africa |  |  |
| *Taphozous mauritianus* |  | EMBALLONURIDAE | 3 | 0 | South Africa |  |  |
| *Rousettus aegyptiacus* |  | PTEROPIDAE | 411 | 1 | South Africa | Lagos bat virus | This report |

^1^ Proposed taxonomic update according to [14,15]

1. Boulger, L.R.; Porterfield, J.S. Isolation of a virus from Nigerian fruit bats. *Trans. R. Soc. Trop. Med. Hyg.* **1958**, *52*, 421–424, doi:10.1016/0035-9203(58)90127-5.

2. Cassel-Beraud, A.M.; Fontenille, D.; Rabetafika, L. Etude bactérienne, virale et parasitaire d’une population de chauves-souris Chaerophon pumila à Anjiro, Madagascar. *Arch. Inst. Pasteur Madagascar* **1989**, *56*, 233–239.

3. Markotter, W. Molecular epidemiology and pathogenesis of Lagos bat virus, a rabies-related virus specific to Africa, University of Pretoria, 2007.

4. Kuzmin, I. V.; Niezgoda, M.; Franka, R.; Agwanda, B.; Markotter, W.; Beagley, J.C.; Urazova, O.Y.; Breiman, R.F.; Rupprecht, C.E. Lagos bat virus in Kenya. *J. Clin. Microbiol.* **2008**, *46*, 1451–1461, doi:10.1128/JCM.00016-08.

5. Kuzmin, I. V.; Mayer, A.E.; Niezgoda, M.; Markotter, W.; Agwanda, B.; Breiman, R.F.; Rupprecht, C.E. Shimoni bat virus, a new representative of the Lyssavirus genus. *Virus Res.* **2010**, *149*, 197–210, doi:10.1016/j.virusres.2010.01.018.

6. Dzikwi, A.A.; Kuzmin, I.I.; Umoh, J.U.; Kwaga, J.K.P.; Ahmad, A.A.; Rupprecht, C.E. Evidence of Lagos Bat Virus circulation among Nigerian fruit bats. *J. Wildl. Dis.* **2010**, *46*, 267–271, doi:10.7589/0090-3558-46.1.267.

7. Hayman, D.T.S.; Fooks, A.R.; Rowcliffe, J.M.; McCrea, R.; Restif, O.; Baker, K.S.; Horton, D.L.; Suu-Ire, R.; Cunningham, A.A.; Wood, J.L.N. Endemic Lagos bat virus infection in Eidolon helvum. *Epidemiol. Infect.* **2012**, *140*, 2163–2171, doi:10.1017/S0950268812000167.

8. Kia, G.S.; Kuzmin, I.I.; Umoh, J.U.; Kwaga, J.K.; Kazeem, H.M.; Osinubi, M.O.; Rupprecht, C.E. Detection of some lyssaviruses from fruigivorous and insectivorous bats in Nigeria. *Online J. Public Health Inform.* **2014**, *6*, 2579, doi:10.5210/ojphi.v6i1.5071.

9. Freuling, C.M.; Binger, T.; Beer, M.; Adu-Sarkodie, Y.; Schatz, J.; Fischer, M.; Hanke, D.; Hoffmann, B.; Höper, D.; Mettenleiter, T.C.; et al. Lagos bat virus transmission in an Eidolon helvum bat colony, Ghana. *Virus Res.* **2015**, *210*, 42–45, doi:10.1016/j.virusres.2015.07.009.

10. Mélade, J.; McCulloch, S.; Ramasindrazana, B.; Lagadec, E.; Turpin, M.; Pascalis, H.; Goodman, S.M.; Markotter, W.; Dellagi, K. Serological evidence of lyssaviruses among bats on Southwestern Indian Ocean Islands. *PLoS One* **2016**, *11*, e0160553, doi:10.1371/journal.pone.0160553.

11. Kalemba, L.N.; Niezgoda, M.; Gilbert, A.T.; Doty, J.B.; Wallace, R.M.; Malekani, J.M.; Carroll, D.S. Exposure to lyssaviruses in bats of the Democratic Republic of the Congo. *J. Wildl. Dis.* **2017**, *53*, 408–410, doi:10.7589/2016-06-122.

12. Suu-Ire, R.; Fooks, A.; Banyard, A.; Selden, D.; Amponsah-Mensah, K.; Riesle, S.; Ziekah, M.; Ntiamoa-Baidu, Y.; Wood, J.; Cunningham, A. Lagos bat virus infection dynamics in free-ranging straw-colored fruit bats (Eidolon helvum). *Trop. Med. Infect. Dis.* **2017**, *2*, 25, doi:10.3390/tropicalmed2030025.

13. Coertse, J.; Grobler, C.S.; Sabeta, C.T.; Seamark, E.C.J.; Kearney, T.; Paweska, J.T.; Markotter, W. Lyssaviruses in insectivorous bats, South Africa, 2003–2018. *Emerg. Infect. Dis.* **2020**, *26*, 3056–3060, doi:10.3201/eid2612.203592.

14. *African Chiroptera Report*; Van Cakenberghe, V., Seamark, E.C.., Eds.; AfricanBats NPC, 2020; ISBN 9780323609845.

15. Monadjem, A.; Demos, T.C.; Dalton, D.L.; Webala, P.W.; Musila, S.; Kerbis Peterhans, J.C.; Patterson, B.D. A revision of pipistrelle-like bats (Mammalia: Chiroptera: Vespertilionidae) in East Africa with the description of new genera and species. *Zool. J. Linn. Soc.* **2020**, 1–33, doi:10.1093/zoolinnean/zlaa087.
