# Supplementary material for: Lagos Bat Virus, an Under-Reported Rabies-Related Lyssavirus
Source: Viruses. 2021 Mar 29;13(4):576. doi: 10.3390/v13040576 (PMC8067007; doi:10.3390/v13040576)
Supplement: Supplementary file 1 [file viruses-13-00576-s001.zip › Table S2. Details of serological surveillance studies for Lagos bat virus in Africa.docx]

Table S2. Details of serological surveillance studies for Lagos bat virus in Africa

| Animal species | Proposed taxonomic update^1^ | Family | Country | Method^2^ | Cut-off value | Number tested | Number postive | Isolate | Reference |
| --- | --- | --- | --- | --- | --- | --- | --- | --- | --- |
| *Eidolon helvum* |  | PTEROPIDAE | Nigeria | MNT | 1:10 | 105 | 24 | Nigeria 1956 | [1] |
| *Micropteropus pusillus* | *Epomophorus pusillus* | PTEROPIDAE | Nigeria | MNT | 1:10 | 21 | 0 | Nigeria 1956 |  |
| *Rhinolophus alcyone* |  | RHINOLOPHIDAE | Nigeria | MNT | 1:10 | 2 | 0 | Nigeria 1956 |  |
| *Epomophorus gambianus* |  | PTEROPIDAE | Nigeria | MNT | 1:10 | 12 | 3 | Nigeria 1956 |  |
| *Eidolon helvum* |  | PTEROPIDAE | Ghana | mRFFIT | 1:10 | 567 | 298 | Ghana 2013 | [2] |
| *Eidolon helvum* |  | PTEROPIDAE | Ghana | mRFFIT | 1:10 | 40 | 0 | Nigeria 1956 |  |
| *Eidolon helvum* |  | PTEROPIDAE | Ghana | FAVN | 1:9 | 57 | 21 | NS^3^ | [3] |
| *Epomophorus gambianus* |  | PTEROPIDAE | Ghana | FAVN | 1:9 | 91 | 3 | NS |  |
| *Epomops buettikoferi* |  | PTEROPIDAE | Ghana | FAVN | 1:9 | 6 | 2 | NS |  |
| *Epomops franqueti* |  | PTEROPIDAE | Ghana | FAVN | 1:9 | 31 | 0 | NS |  |
| *Hypsignathus monstrosus* |  | PTEROPIDAE | Ghana | FAVN | 1:9 | 5 | 0 | NS |  |
| *Nanonycteris veldkampii* |  | VESPERTILLIONIDAE | Ghana | FAVN | 1:9 | 4 | 0 | NS |  |
| *Eidolon helvum* |  | PTEROPIDAE | Ghana | mFAVN | 1:9 | 1162 | 358 | Nigeria 1956 | [4] |
| *Eidolon helvum* |  | PTEROPIDAE | DRC | MNT | 1:10 | 18 | 6 | Afr1999 | [5] |
| *Micropteropus pusillus* | *Epomophorus pusillus* | PTEROPIDAE | DRC | MNT | 1:10 | 1 | 0 | Afr1999 |  |
| *Chaerephon pumilus* | *Mops pumilus* | MOLOSSIDAE | DRC | MNT | 1:10 | 23 | 0 | Afr1999 |  |
| *Chaerephon sp.* | *Mops sp.* | MOLOSSIDAE | DRC | MNT | 1:10 | 20 | 0 | Afr1999 |  |
| *Glauconycteris argentata* |  | VESPERTILLIONIDAE | DRC | MNT | 1:10 | 1 | 0 | Afr1999 |  |
| *Hipposideros fuliginosus* |  | HIPPOSIDERIDAE | DRC | MNT | 1:10 | 20 | 0 | Afr1999 |  |
| *Macronycteris (Hipposideros) gigas* |  | HIPPOSIDERIDAE | DRC | MNT | 1:10 | 1 | 0 | Afr1999 |  |
| *Hypsignathus monstrosus* |  | PTEROPIDAE | DRC | MNT | 1:10 | 2 | 0 | Afr1999 |  |
| *Megaglossus woermanni* |  | PTEROPIDAE | DRC | MNT | 1:10 | 8 | 0 | Afr1999 |  |
| *Mimetillus moloneyi* |  | VESPERTILLIONIDAE | DRC | MNT | 1:10 | 1 | 0 | Afr1999 |  |
| *Miniopterus sp.* |  | MINIOPTERIDAE | DRC | MNT | 1:10 | 38 | 0 | Afr1999 |  |
| *Mops condylurus* |  | MOLOSSIDAE | DRC | MNT | 1:10 | 31 | 0 | Afr1999 |  |
| *Myonycteris torquata* |  | PTEROPIDAE | DRC | MNT | 1:10 | 6 | 2 | Afr1999 |  |
| *Myotis sp.* |  | VESPERTILLIONIDAE | DRC | MNT | 1:10 | 1 | 0 | Afr1999 |  |
| *Neoromicia sp.* |  | VESPERTILLIONIDAE | DRC | MNT | 1:10 | 1 | 0 | Afr1999 |  |
| *Pipistrellus sp.* |  | VESPERTILLIONIDAE | DRC | MNT | 1:10 | 35 | 0 | Afr1999 |  |
| *Rhinolophus sp.* |  | RHINOLOPHIDAE | DRC | MNT | 1:10 | 1 | 0 | Afr1999 |  |
| *Scotophilus dinganii* |  | VESPERTILLIONIDAE | DRC | MNT | 1:10 | 2 | 0 | Afr1999 |  |
| *Eidolon helvum* |  | PTEROPIDAE | Nigeria | RFFIT | NS | 70 | 21 | NS | [6] |
| *Epomophorus gambianus* |  | PTEROPIDAE | Nigeria | RFFIT | NS | 1 | 0 | NS |  |
| *Chaerephon pumilus* | *Mops pumilus* | MOLOSSIDAE | Nigeria | RFFIT | NS | 4 | 1 | NS |  |
| *Nycteris macrotis* |  | NYCTERIDAE | Nigeria | RFFIT | NS | 1 | 0 | NS |  |
| *Eidolon helvum* |  | PTEROPIDAE | Kenya | MNT | 1:10 | 9 | 4 | KE576 | [7] |
| *Hipposideros commersoni* | *Macronycteris vitattus* |  | Kenya | MNT | 1:10 | 99 | 5 | KE576 |  |
| *Hipposideros sp.* |  | HIPPOSIDERIDAE | Kenya | MNT | 1:10 | 22 | 0 | KE576 |  |
| *Triaenops persicus* | *Triaenops afer* | RHINONYCTERIDAE | Kenya | MNT | 1:10 | 8 | 0 | KE576 |  |
| *Cardioderma cor* |  | MEGADERMITIDAE | Kenya | MNT | 1:10 | 3 | 0 | KE576 |  |
| *Chaerephon sp.* | *Mops sp.* | MOLOSSIDAE | Kenya | MNT | 1:10 | 81 | 0 | KE576 |  |
| *Coleura afra* |  | EMBALLONURIDAE | Kenya | MNT | 1:10 | 24 | 0 | KE576 |  |
| *Miniopterus sp.* |  | MINIOPTERIDAE | Kenya | MNT | 1:10 | 235 | 2 | KE576 |  |
| *Neoromicia sp.* |  | VESPERTILLIONIDAE | Kenya | MNT | 1:10 | 47 | 0 | KE576 |  |
| *Nycteris sp.* |  | NYCTERIDAE | Kenya | MNT | 1:10 | 13 | 0 | KE576 |  |
| *Otomops martiensseni* |  | MOLOSSIDAE | Kenya | MNT | 1:10 | 35 | 0 | KE576 |  |
| *Pipistrellus sp.* |  | VESPERTILLIONIDAE | Kenya | MNT | 1:10 | 4 | 0 | KE576 |  |
| *Rhinolophus sp.* |  | RHINOLOPHIDAE | Kenya | MNT | 1:10 | 41 | 0 | KE576 |  |
| *Scotoecus sp.* |  | VESPERTILLIONIDAE | Kenya | MNT | 1:10 | 3 | 0 | KE576 |  |
| *Scotophilus dingani* |  | VESPERTILLIONIDAE | Kenya | MNT | 1:10 | 10 | 0 | KE576 |  |
| *Epomophorus sp.* |  | PTEROPIDAE | Kenya | MNT | 1:10 | 56 | 1 | KE576 |  |
| *Rousettus aegypticaus* |  | PTEROPIDAE | Kenya | MNT | 1:10 | 79 | 37 | KE576 |  |
| *Miniopterus griveaudi* |  | MINIOPTERIDAE | Anjouan | MNT | 1:25 | 2 | 0 | Afr1999 | [8] |
| *Chaerephon pusillus* | *Mops pusillus* | MOLOSSIDAE | Anjouan | MNT | 1:25 | 19 | 2 | Afr1999 |  |
| *Hipposideros commersoni* | *Macronycteris commersoni* | HIPPOSIDERIDAE | Madagascar | MNT | 1:25 | 6 | 0 | Afr1999 |  |
| *Miniopterus cf. ambohitrensis* |  | MINIOPTERIDAE | Madagascar | MNT | 1:25 | 17 | 0 | Afr1999 |  |
| *Miniopterus gleni* |  | MINIOPTERIDAE | Madagascar | MNT | 1:25 | 4 | 0 | Afr1999 |  |
| *Miniopterus griveaudi* |  | MINIOPTERIDAE | Madagascar | MNT | 1:25 | 33 | 2 | Afr1999 |  |
| *Miniopterus mahafaliensis* |  | MINIOPTERIDAE | Madagascar | MNT | 1:25 | 11 | 0 | Afr1999 |  |
| *Miniopterus sororculus* |  | MINIOPTERIDAE | Madagascar | MNT | 1:25 | 3 | 0 | Afr1999 |  |
| *Chaerephon atsinanana* | *Mops atsinanana* | MOLOSSIDAE | Madagascar | MNT | 1:25 | 16 | 0 | Afr1999 |  |
| *Chaerephon leucogaster* | *Mops leucogaster* | MOLOSSIDAE | Madagascar | MNT | 1:25 | 28 | 0 | Afr1999 |  |
| *Mops leucostigma* |  | MOLOSSIDAE | Madagascar | MNT | 1:25 | 20 | 2 | Afr1999 |  |
| *Mops midas* |  | MOLOSSIDAE | Madagascar | MNT | 1:25 | 7 | 0 | Afr1999 |  |
| *Mormopterus jugalaris* |  | MOLOSSIDAE | Madagascar | MNT | 1:25 | 58 | 3 | Afr1999 |  |
| *Otomops madagascariensis* |  | MOLOSSIDAE | Madagascar | MNT | 1:25 | 20 | 3 | Afr1999 |  |
| *Eidolon dupreanum* |  | PTEROPIDAE | Madagascar | MNT | 1:25 | 9 | 0 | Afr1999 |  |
| *Triaenops menamena* |  | RHINONYCTERIDAE | Madagascar | MNT | 1:25 | 11 | 2 | Afr1999 |  |
| *Myotis goudoti* |  | VESPERTILLIONIDAE | Madagascar | MNT | 1:25 | 11 | 4 | Afr1999 |  |
| *Mormopterus acetabulosus* |  | MOLOSSIDAE | Mauritius | MNT | 1:25 | 31 | 5 | Afr1999 |  |
| *Chaerephon pusillus* | *Mops pusillus* | MOLOSSIDAE | Mayotte | MNT | 1:25 | 3 | 1 | Afr1999 |  |
| *Mormopterus francoismoutoui* |  | MOLOSSIDAE | La Reunion | MNT | 1:25 | 121 | 3 | Afr1999 |  |
| *Pteropus niger* |  | PTEROPIDAE | Mauritius | MNT | 1:25 | 36 | 2 | Afr1999 |  |
| *Pteropus rufus* |  | PTEROPIDAE | Madagascar | MNT | 1:25 | 12 | 0 | Afr1999 |  |
| *Pteropus seychellensis* |  | PTEROPIDAE | Mahe (Seychelles) | MNT | 1:25 | 40 | 4 | Afr1999 |  |
| *Pteropus seychellensis* |  | PTEROPIDAE | Mayotte | MNT | 1:25 | 19 | 2 | Afr1999 |  |
| *Rousettus madagascariensis* |  | PTEROPIDAE | Madagascar | MNT | 1:25 | 35 | 7 | Afr1999 |  |
| *Eidolon dupreanum* |  | PTEROPIDAE | Madagascar | RFFIT | >42 | 50 | 12 | NS | [9] |
| *Pteropus rufus* |  | PTEROPIDAE | Madagascar | RFFIT | >42 | 28 | 1 | NS |  |
| *Eidolon helvum* |  | PTEROPIDAE | Ghana | mFAVN | NS | 304 | 136 | Nigeria 1956 | [10] |
| *Eidolon helvum* |  | PTEROPIDAE | Ghana | mFAVN & PNA | 1:40 | 183 | 103 | Nigeria 1956 | [11] |
| *Hypsignathus monstrosus* |  | PTEROPIDAE | Ghana | mFAVN & PNA | 1:40 | 1 | 1 | Nigeria 1956 |  |

^1^ Proposed taxonomic update according to [12,13]

^2^ MNT: micro-neutralization test, RFFIT: rapid fluorescent focus inhibition test, FAVN: fluorescent antibody virus neutralization test, mRFFIT: modified rapid fluorescent focus inhibition test, mFAVN: modified fluorescent antibody virus neutralization test, PNA: pseudotyped neutralisation assay

^3^ NS: Not specified

1. Dzikwi, A.A.; Kuzmin, I.I.; Umoh, J.U.; Kwaga, J.K.P.; Ahmad, A.A.; Rupprecht, C.E. Evidence of Lagos Bat Virus circulation among Nigerian fruit bats. *J. Wildl. Dis.* **2010**, *46*, 267–271, doi:10.7589/0090-3558-46.1.267.

2. Freuling, C.M.; Binger, T.; Beer, M.; Adu-Sarkodie, Y.; Schatz, J.; Fischer, M.; Hanke, D.; Hoffmann, B.; Höper, D.; Mettenleiter, T.C.; et al. Lagos bat virus transmission in an Eidolon helvum bat colony, Ghana. *Virus Res.* **2015**, *210*, 42–45, doi:10.1016/j.virusres.2015.07.009.

3. Hayman, D.T.S.; Fooks, A.R.; Horton, D.; Suu-Ire, R.; Breed, A.C.; Cunningham, A.A.; Wood, J.L.N. Antibodies against Lagos Bat Virus in Megachiroptera from West Africa. *Emerg. Infect. Dis.* **2008**, *14*, 926–928, doi:10.3201/eid1406.071421.

4. Hayman, D.T.S.; Fooks, A.R.; Rowcliffe, J.M.; McCrea, R.; Restif, O.; Baker, K.S.; Horton, D.L.; Suu-Ire, R.; Cunningham, A.A.; Wood, J.L.N. Endemic Lagos bat virus infection in Eidolon helvum. *Epidemiol. Infect.* **2012**, *140*, 2163–2171, doi:10.1017/S0950268812000167.

5. Kalemba, L.N.; Niezgoda, M.; Gilbert, A.T.; Doty, J.B.; Wallace, R.M.; Malekani, J.M.; Carroll, D.S. Exposure to lyssaviruses in bats of the Democratic Republic of the Congo. *J. Wildl. Dis.* **2017**, *53*, 408–410, doi:10.7589/2016-06-122.

6. Kia, G.S.; Kuzmin, I.I.; Umoh, J.U.; Kwaga, J.K.; Kazeem, H.M.; Osinubi, M.O.; Rupprecht, C.E. Detection of some lyssaviruses from fruigivorous and insectivorous bats in Nigeria. *Online J. Public Health Inform.* **2014**, *6*, 2579, doi:10.5210/ojphi.v6i1.5071.

7. Kuzmin, I. V.; Turmelle, A.S.; Agwanda, B.; Markotter, W.; Niezgoda, M.; Breiman, R.F.; Rupprecht, C.E. Commerson’s leaf-nosed bat (Hipposideros commersoni) is the likely reservoir of Shimoni bat virus. *Vector-Borne Zoonotic Dis.* **2011**, *11*, 1465–1470, doi:10.1089/vbz.2011.0663.

8. Mélade, J.; McCulloch, S.; Ramasindrazana, B.; Lagadec, E.; Turpin, M.; Pascalis, H.; Goodman, S.M.; Markotter, W.; Dellagi, K. Serological evidence of lyssaviruses among bats on Southwestern Indian Ocean Islands. *PLoS One* **2016**, *11*, e0160553, doi:10.1371/journal.pone.0160553.

9. Reynes, J.-M.; Andriamandimby, S.F.; Razafitrimo, G.M.; Razainirina, J.; Jeanmaire, E.M.; Bourhy, H.; Heraud, J.-M. Laboratory surveillance of rabies in humans, domestic animals, and bats in Madagascar from 2005 to 2010. *Adv. Prev. Med.* **2011**, *2011*, 1–6, doi:10.4061/2011/727821.

10. Suu-Ire, R.; Fooks, A.; Banyard, A.; Selden, D.; Amponsah-Mensah, K.; Riesle, S.; Ziekah, M.; Ntiamoa-Baidu, Y.; Wood, J.; Cunningham, A. Lagos bat virus infection dynamics in free-ranging straw-colored fruit bats (Eidolon helvum). *Trop. Med. Infect. Dis.* **2017**, *2*, 25, doi:10.3390/tropicalmed2030025.

11. Wright, E.; Hayman, D.T.S.; Vaughan, A.; Temperton, N.J.; Wood, J.L.N.; Cunningham, A.A.; Suu-Ire, R.; Weiss, R.A.; Fooks, A.R. Virus neutralising activity of African fruit bat (Eidolon helvum) sera against emerging lyssaviruses. *Virology* **2010**, *408*, 183–189, doi:10.1016/j.virol.2010.09.014.

12. *African Chiroptera Report*; Van Cakenberghe, V., Seamark, E.C.., Eds.; AfricanBats NPC, 2020; ISBN 9780323609845.

13. Monadjem, A.; Demos, T.C.; Dalton, D.L.; Webala, P.W.; Musila, S.; Kerbis Peterhans, J.C.; Patterson, B.D. A revision of pipistrelle-like bats (Mammalia: Chiroptera: Vespertilionidae) in East Africa with the description of new genera and species. *Zool. J. Linn. Soc.* **2020**, 1–33, doi:10.1093/zoolinnean/zlaa087.
