# Supplementary material for: Lagos Bat Virus, an Under-Reported Rabies-Related Lyssavirus
Source: Viruses. 2021 Mar 29;13(4):576. doi: 10.3390/v13040576 (PMC8067007; doi:10.3390/v13040576)
Supplement: Supplementary file 1 [file viruses-13-00576-s001.zip › Table S3. Details of lyssavirus sequences used for phylogenetic analysis.docx]

Table S3. Details of lyssavirus sequences used for phylogenetic analysis

| Genbank accession number | Lyssavirus | Phylogroup | Host | Year | Country |
| --- | --- | --- | --- | --- | --- |
| KP723638 | Rabies virus | I | *Canis simensis* | 2014 | Ethiopia |
| KT336437 | Rabies virus | I | *Canis lupus familiaris* | 2012 | South Africa |
| HQ450386 | Rabies virus | I | *Canis familiaris* | Not specified | Mexico |
| AY352493 | Rabies virus | I | *Homo sapiens* | Not specified | India |
| KF977826 | Rabies virus | I | *Homo sapiens* | 2011 | Central African Republic |
| AF374721 | Rabies virus | I | *Canis familiaris* | Not specified | India |
| GU358653 | Rabies virus | I | *Canis familiaris* | 1994 | China |
| KM594025 | Rabies virus | I | *Callithrix jacchus* | 2007 | Brazil |
| AF351847 | Rabies virus | I | *Desmodus rotundus* | 1986 | Brazil |
| AF351850 | Rabies virus | I | Insectivorous bat | 1988 | Chile |
| NC031988 | Gannoruwa bat lyssavirus | I | *Pteropus giganteus* | 2015 | Sri Lanka |
| AY573937 | Australian bat lyssavirus | I | *Saccolaimus flaviventris* | 1997 | Australia |
| AY573965 | Australian bat lyssavirus | I | *Saccolaimus flaviventris* | 1997 | Australia |
| NC003243 | Australian bat lyssavirus | I | *Saccolaimus flaviventris* | 1996 | Australia |
| AF418014 | Australian bat lyssavirus | I | *Homo sapiens* | 1998 | Australia |
| AY573964 | Australian bat lyssavirus | I | *Pteropus alecto* | 1997 | Australia |
| KT868955 | Australian bat lyssavirus | I | *Pteropus alecto* | 2014 | Australia |
| KT868956 | Australian bat lyssavirus | I | *Pteropus scapulatus* | 2014 | Australia |
| AY573963 | Australian bat lyssavirus | I | *Pteropus alecto* | 1998 | Australia |
| NC020808 | Aravan virus | I | *Myotis blythi* | 1991 | Kyrgyzstan |
| MF960865 | Kotalahti bat lyssavirus | I | *Myotis brandtii* | 2017 | Finland |
| MF043188 | Bokeloh bat lyssavirus | I | *Myotis nattereri* | 2013 | France |
| KC169985 | Bokeloh bat lyssavirus | I | *Myotis nattereri* | 2012 | France |
| KC169985 | Bokeloh bat lyssavirus | I | *Myotis nattereri* | 2012 | France |
| NC05385 | Khujand virus | I | *Myotis mystacinus* | 2001 | Tajikistan |
| AY863408 | European bat lyssavirus 2 | I | Not reported | 2002 | Switzerland |
| AY863407 | European bat lyssavirus 2 | I | *Myotis daubentonii* | 1993 | Switzerland |
| KF155004 | European bat lyssavirus 2 | I | *Myotis daubentonii* | 2004 | United Kingdom |
| NC009528 | European bat lyssavirus 2 | I | *Homo sapiens* | 2002 | United Kingdom |
| AY863405 | European bat lyssavirus 2 | I | *Myotis dasycneme* | 1989 | Netherlands |
| KY688152 | European bat lyssavirus 2 | I | *Myotis dasycneme* | 1993 | Netherlands |
| EU293114 | European bat lyssavirus 2 | I | *Myotis dasycneme* | 1986 | Netherlands |
| MF472709 | Taiwan bat lyssavirus | I | *Pipistrellus abramus* | 2017 | Taiwan |
| MF472710 | Taiwan bat lyssavirus | I | *Pipistrellus abramus* | 2016 | Taiwan |
| NC020809 | Irkut virus | I | *Murina leucogaster* | 2002 | Russia |
| EU293119 | Duvenhage virus | I | *Homo sapiens* | 1971 | South Africa |
| EU293120 | Duvenhage virus | I | *Miniopterus sp* | 1981 | South Africa |
| KC866301 | Duvenhage virus | I | *Nycteris thebaica* | 2012 | South Africa |
| EU623444 | Duvenhage virus | I | *Homo sapiens* | 2006 | South Africa |
| JN986749 | Duvenhage virus | I | *Homo sapiens* | 2007 | Netherlands (imported from Kenya) |
| KP241939 | European bat lyssavirus 1 | I | *Eptesicus isabellinus* | 2007 | Spain |
| EU626552 | European bat lyssavirus 1 | I | *Felis catus* | 2007 | France |
| KF155003 | European bat lyssavirus 1 | I | *Eptesicus serotinus* | 1986 | Denmark |
| AY863382 | European bat lyssavirus 1 | I | *Eptesicus serotinus* | 2001 | Slovakia |
| AY863364 | European bat lyssavirus 1 | I | *Eptesicus serotinus* | 2000 | Netherlands |
| NC009527 | European bat lyssavirus 1 | I | *Eptesicus serotinus* | 1968 | Germany |
| EF157976 | European bat lyssavirus 1 | I | *Eptesicus serotinus* | 1968 | Germany |
| NC025365 | Shimoni bat virus | II | *Macronycteris vittatus* | 2009 | Kenya |
| FJ465413 | Mokola virus | II | *Felis catus* | 1997 | South Africa |
| JN944637 | Mokola virus | II | *Felis catus* | 1997 | South Africa |
| FJ465410 | Mokola virus | II | *Felis catus* | 1998 | South Africa |
| KP899612 | Mokola virus | II | *Felis catus* | 2014 | South Africa |
| KP899610 | Mokola virus | II | *Felis catus* | 2012 | South Africa |
| KP899611 | Mokola virus | II | *Felis catus* | 2012 | South Africa |
| KC218934 | Mokola virus | II | *Felis catus* | 2008 | South Africa |
| FJ465412 | Mokola virus | II | *Felis catus* | 2006 | South Africa |
| FJ465414 | Mokola virus | II | *Felis catus* | 1996 | South Africa |
| KF155008 | Mokola virus | II | *Felis catus* | 1996 | South Africa |
| FJ465415 | Mokola virus | II | *Felis catus* | 1995 | South Africa |
| KF155005 | Mokola virus | II | *Crocidura sp.* | 1968 | Nigeria |
| EU293117 | Mokola virus | II | *Crocidura sp.* | 1974 | Cameroon |
| KF155007 | Mokola virus | II | *Felis catus* | 1993 | Zimbabwe |
| FJ465418 | Mokola virus | II | *Felis catus* | 1981 | Zimbabwe |
| FJ465417 | Mokola virus | II | *Felis catus* | 1981 | Zimbabwe |
| KF155006 | Mokola virus | II | *Felis catus* | 1982 | Zimbabwe |
| KC218932 | Mokola virus | II | *Felis catus* | 1981 | Zimbabwe |
| EU293118 | Mokola virus | II | *Laphuromys sikapusi* | 1981 | Central African Republic |
| EF547447 | Lagos bat virus | II | *Rousettus aegyptiacus* | 1999 | France (imported from Africa) |
| LN849915 | Lagos bat virus | II | *Eidolon helvum* | 2013 | Ghana |
| EU293108 | Lagos bat virus | II | *Eidolon helvum* | 1985 | Senegal |
| EU259198 | Lagos bat virus | II | *Eidolon helvum* | 2007 | Kenya |
| GU170202 | Lagos bat virus | II | *Rousettus aegyptiacus* | 2008 | Kenya |
| JX901139 | Lagos bat virus | II | *Rousettus aegyptiacus* | 2010 | Kenya |
| MW358036 | Lagos bat virus | II | *Rousettus aegyptiacus* | 2018 | South Africa |
| EU293110 | Lagos bat virus | II | *Eidolon helvum* | 1956 | Nigeria |
| EF547449 | Lagos bat virus | II | *Micropterus pusillus* | 1974 | Central African Republic |
| EF547450 | Lagos bat virus | II | *Felis catus* | 1986 | Zimbabwe |
| DQ499945 | Lagos bat virus | II | *Epomophorus wahlbergi* | 2004 | South Africa |
| EF547456 | Lagos bat virus | II | *Epomophorus wahlbergi* | 1980 | South Africa |
| AY333110 | Lagos bat virus | II | *Canis familiaris* | 1990 | Ethiopia |
| EF547457 | Lagos bat virus | II | *Epomophorus wahlbergi* | 1980 | South Africa |
| EF547454 | Lagos bat virus | II | *Epomophorus wahlbergi* | 1980 | South Africa |
| DQ499948 | Lagos bat virus | II | *Atilax paludinosus* | 2004 | South Africa |
| MH643892 | Lagos bat virus | II | *Epomophorus wahlbergi* | 2018 | South Africa |
| MH643890 | Lagos bat virus | II | *Epomophorus wahlbergi* | 2013 | South Africa |
| MH643893 | Lagos bat virus | II | *Epomophorus wahlbergi* | 2014 | South Africa |
| HM179509 | Lagos bat virus | II | *Epomophorus wahlbergi* | 2008 | South Africa |
| DQ499944 | Lagos bat virus | II | *Epomophorus wahlbergi* | 2003 | South Africa |
| EF547452 | Lagos bat virus | II | *Epomophorus wahlbergi* | 2006 | South Africa |
| KP994622 | Lagos bat virus | II | *Felis catus* | 2013 | South Africa |
| MW358036 | Lagos bat virus | II | *Epomophorus wahlbergi* | 2018 | South Africa |
| KP994623 | Lagos bat virus | II | *Epomophorus wahlbergi* | 2014 | South Africa |
| MH643891 | Lagos bat virus | II | *Epomophorus wahlbergi* | 2017 | South Africa |
| NC018629 | Ikoma lyssavirus |  | *Civettictis civetta* | 2009 | Tanzania |
| NC031955 | Lleida bat lyssavirus |  | *Miniopterus schreibersii* | 2011 | Spain |
| NC025377 | West Caucasian bat virus |  | *Miniopterus schreibersii* | 2002 | Russia |
| MT364249 | Matlo bat lyssavirus |  | *Miniopterus natalensis* | 2015 | South Africa |
| MT364250 | Matlo bat lyssavirus |  | *Miniopterus natalensis* | 2016 | South Africa |
